# Neurological Manifestations of IgG4-Related Disease

**DOI:** 10.1007/s11940-017-0450-9

**Published:** 2017-04-03

**Authors:** Bernardo Baptista, Alina Casian, Harsha Gunawardena, David D’Cruz, Claire M. Rice

**Affiliations:** 1 Department of Internal Medicine, Hospital da Luz, Lisbon, Portugal; 2 Louise Coote Unit, Guy’s and St Thomas NHS Foundation Trust, London, UK; 3 Department of Rheumatology, Brunel Building, Southmead Hospital, Bristol, UK; 4 Musculoskeletal Research Unit, Learning and Research Building, University of Bristol, Southmead Hospital, Bristol, BS10 5NB UK; 5 Division of Immunology, Infection and Inflammatory Diseases, King’s College London, New Hunt’s House, Guy’s Campus, Great Maze Pond, London, SE1 1UL UK; 6 School of Clinical Sciences, Level 1, Learning and Research Building, University of Bristol, Southmead Hospital, Bristol, BS10 5NB UK; 7 Department of Neurology, Brunel Building, Southmead Hospital, Bristol, UK

**Keywords:** IgG4-related disease, Pachymeningitis, Hypophysitis, Pseudotumour, Neuropathy

## Abstract

IgG4-related disease (IgG4-RD) is a multisystem inflammatory disorder. Early recognition of IgG4-RD is important to avoid permanent organ dysfunction and disability. Neurological involvement by IgG4-RD is relatively uncommon, but well recognised—hypertrophic pachymeningitis and hypophysitis are the most frequent manifestations. Although the nervous system may be involved in isolation, this more frequently occurs in conjunction with involvement of other systems. Elevated circulating levels of IgG4 are suggestive of the condition, but these are not pathognomonic and exclusion of other inflammatory disorders including vasculitis is required. Wherever possible, a tissue diagnosis should be established. The characteristic histopathological changes include a lymphoplasmacytoid infiltrate, storiform fibrosis and obliterative phlebitis. IgG4-RD typically responds well to treatment with glucocorticoids, although relapse is relatively common and treatment with a steroid-sparing agent or rituximab may be required. Improved understanding of the pathogenesis of IgG4-RD is likely to lead to the development of more specific disease treatments in the future.

## Introduction

IgG4-related disease (IgG4-RD) is a systemic, immune-mediated fibro-inflammatory disease of unknown cause, characterised by unique pathological features involving a wide variety of organs [1, 2••, 3, 4].

Once regarded as an isolated single-organ disease, IgG4-related disease is now recognised as a single multisystem disorder which can affect virtually any organ system (Table 1) [5•, 6]. Clinically, enlargement of the affected organ(s) may be accompanied by high serum levels of IgG4, and the histology shows the classic triad—infiltration of IgG4-bearing plasmacytes, storiform fibrosis and obliterative phlebitis [5•, 7]. Its protean manifestations mean it can mimic many other conditions, including neoplastic, infectious and other inflammatory diseases. These require exclusion if treatment for this eminently treatable condition is to be optimised [8, 9•, 10••].

**Table 1:** Isolated tissue involvement by IgG4-RD is now recognised to occur as part of a multisystem disorder

Disease name	Target organ
IgG4-related orbital disease	Orbits and periorbital tissue
IgG4-related sialadenitis (Mikulicz’s disease, Küttner’s tumour)	Salivary, lacrimal and submandibular glands
IgG4-related thyroiditis (Riedel’s thyroiditis)	Thyroid
IgG4-related sinusitis/midline destructive lesion/pharyngitis	Ear, nose and throat
IgG4-related lung disease	Lungs
IgG4-related pleural disease	Pleura
IgG4-related mediastinitis	Mediastinum
IgG4-related mastitis	Breast
IgG4-related periaortitis	Aorta
IgG4-related retroperitoneal fibrosis (Ormund’s disease)	Retroperitoneum
IgG4-related cardiac disease	Heart and pericardium
IgG4-related sclerosing mesenteritis	Mesentery
IgG4-related autoimmune pancreatitis (type 1)	Pancreas
IgG4-related sclerosing cholangitis	Bile ducts
IgG4-related hepatitis	Liver
IgG4-related gastrointestinal disease	Gastrointestinal tract
IgG4-related interstitial nephritis/glomerulonephritis (idiopathic hypocomplementemic tubulointerstitial nephritis with extensive tubulointerstitial deposits)	Kidney
IgG4-related prostatitis	Prostate
IgG4-related epididymo-orchitis	Testis
IgG4-related hypophysitis	Hypophysis
IgG4-related pachymeningitis	Dura mater
IgG4-related neuropathy	Peripheral nerves
IgG4-related lymphadenopathy	Lymph nodes
IgG4-related skin disease	Skin
IgG4-related disease of the bone	Bone

The organs commonly affected by IgG4-RD are the pancreas (autoimmune pancreatitis), bile ducts (sclerosing cholangitis), retroperitoneum (retroperitoneal fibrosis), salivary glands (sclerosing sialadenitis) and lacrimal glands (dacryoadenitis). The nervous system is less commonly involved, although a variety of manifestations are recognised [11, 12].

The first report of central nervous system (CNS) involvement by IgG4-RD occurred in the context of hypophysitis [13], but since then, hypertrophic pachymeningitis has also been recognised to occur as part of the IgG4-RD spectrum, and IgG4-RD may account for a substantial percentage of cases previously regarded as idiopathic [14, 15]. Here, we focus on the clinical features, diagnosis and management of the neurological manifestations of IgG4-RD, which, although uncommon, may be a life-threatening manifestation of a treatable disease.

## History

In 1961, Sarles et al. raised the possibility that chronic inflammatory sclerosis of the pancreas was a distinct clinical entity [16]. However, it was not until 1995 that Yoshida et al. suggested the concept of autoimmune pancreatitis based on the clinical features of serum autoantibodies, hypergammaglobulinemia, occasional association with other autoimmune diseases, histological evidence of lymphoplasmacytic inflammation and fibrosis and a favourable response to glucocorticoid treatment [17]. In 2001, Hamano et al. described high serum IgG4 concentrations in patients with sclerosing pancreatitis, but not in patients with pancreatic carcinoma, non-specific chronic pancreatitis, primary biliary cirrhosis, primary sclerosing cholangitis and normal individuals—a condition now known as type 1 (IgG4-related) autoimmune pancreatitis (AIP) [7]. Although reports had previously reported the coincidence of the number of apparently isolated diseases—for example the case described by Montefusco et al. with sclerosing cholangitis, chronic pancreatitis and Sjögren’s syndrome [18]—the systemic nature of IgG4-RD has only been truly appreciated since Kamisawa et al. proposed the clinicopathological entity ‘IgG4-related autoimmune disease’ with pancreatic, bile duct, retroperitoneal and salivary gland involvement in 2003 [13, 19–29].

## Epidemiology

Few large-scale epidemiological studies of IgG4-RD have been performed, and the majority have focused on AIP in Japanese cohorts [30]. In contrast to other autoimmune diseases, a male predominance of IgG4-RD has been reported, with a peak onset of disease at 61–70 years [31]. In keeping with the more recent and widespread recognition of the condition, four studies describing relatively large cohorts of patients with IgG4-RD have been published within the last 2 years: three retrospective and one prospective, with two originating from the USA, one from China and one from Japan [32, 33, 34••, 35]. The main demographic and clinical findings are summarised in Table 2. In all studies, neurological disease was relatively rare, although cases of pituitary, meningeal and peripheral nervous system involvement were reported. In two smaller European studies, one from Spain and the other one from Italy, pachymeningitis was the only reported neurological manifestation—2/55 (4%) and 3/41 (7%), respectively [36, 37].

**Table 2:** Summary findings of four cohorts of patients with IgG4-RD published within the last 2 years [32, 33, 34••, 35]

	Lin et al. (*n* = 118)	Wallace et al. (*n* = 125)	Inoue et al. (*n* = 235)	Sekiguchi et al. (*n* = 166)
Type of study	Prospective	Retrospective	Retrospective	Retrospective
Age, mean (range, years)	53.1 (19–80)	55.2 (24–83)	67 (35–86)	61 (49–70)
Men/women, *n* (%)	82 (69)/36 (31)	76 (61)/49 (39)	189 (80)/46 (20)	125 (75)/41 (25)
Single organ/≥2 organs (%)	4.2/95.8	38/62	42/58	20/80
Organ involvement, *n* (%)				
Pancreas	45 (38.1)	24 (19.2)	142 (60)	107 (64.5)
Bile ducts	21 (17.8)	12 (9.6)	31 (13)	93 (56)
Lacrimal glands	60 (50.8)	28 (22.4) orbits^a^	53 (23)	14 (8.4)
Salivary glands	76 (64.4)	35 (28)	81 (34)	16 (9.6)
Lymphadenopathy	77 (65.3)	34 (27.2)	34 (14)	29 (17)
Retroperitoneal fibrosis/periaortitis	31 (26.3)	37 (29.6)	57 (24)	24 (14.5)
Kidney	29 (24.6)	15 (12)	54 (23)	21 (12.7)
Lungs	32 (27.1)	22 (17.6)	31 (13)	23 (13.9)
Prostate	29 (24.6)	4 (3.2)	–^a^	3 (1.8)
Pituitary	2 (1.7)	None	–^a^	2 (1.2)
Meninges	None	3 (2.4)	None	None
Peripheral nerves	None	1 (0.8)	–^a^	None

^a^Reported as other sites (3%)—prostate, peripheral nerve, pituitary gland, skin and pericardium

## Pathophysiology

IgG4 is one of the four human IgG subclasses and usually constitutes <5% of IgG in healthy individuals [38]. Despite having over 90% amino acid sequence homology with the other subclasses, IgG4 has a unique structure and function and is generally regarded as non-inflammatory because it does not efficiently engage activating Fc receptors or complement and might be functionally monovalent in vivo [39]. Although IgG4 autoantibodies are known to be pathogenic in a number of diseases including pemphigus vulgaris, thrombotic thrombocytopenic purpura and idiopathic membranous nephropathy, there is growing acceptance that IgG4 itself is unlikely to be pathogenic in IgG4-RD [2••, 40, 41].

Indeed, the effectiveness of B cell depletion therapy in IgG4-RD suggests that B lymphocytes and other cells of this lineage play an important pathological role, probably via their interaction with CD4^+^ cytotoxic T lymphocytes (CTL) serving as effective antigen-presenting cells and/or through the secretion of B cell-derived growth factors [42, 43].

Plasmablasts (CD19^+^CD20^−^CD27^+^CD38^+^) are found in high concentrations in IgG4-RD, regardless of the serum IgG4 concentration, and their number may correlate more strongly with disease activity than IgG4 levels [44]. Circulating oligoclonal plasmablasts demonstrate intense somatic hypermutation, supporting the idea that T helper cell-dependent processes are likely to be important in IgG4-RD pathophysiology [45]. How and why specific B cells are recruited to become clonally expanded IgG4-producing plasmablasts and plasma cells remains unknown, but T follicular helper cells appear to drive the class switch towards IgG4, perhaps through the secretion of IL-4 (amongst other cytokines) [41, 45]. Wenniger et al. recently confirmed highly abundant IgG4^+^ B cells and plasma cell clones (through analysis of the B cell receptor heavy chain) in blood and tissue of patients with active IgG4-related cholangitis; these disappeared with corticosteroid treatment, suggesting that specific B cell responses may be pivotal to the pathogenesis of IgG4-RD [46]. Furthermore, the presence of oligoclonal IgG4 bands in the cerebrospinal fluid (CSF) of subjects with IgG4-related hypertrophic pachymeningitis supports the concept of an antigen-driven immune response [47, 48].

On the other hand, CD4^+^ T cells have been shown to be dispersed throughout IgG4-RD lesions and to be the most abundant cell within affected tissues [4, 5•, 12, 49]. The recent characterisation of a clonally expanded population of CD4^+^ CTL in both the peripheral blood and fibrotic lesions of IgG4-RD patients suggests that these cells are indeed central to the disease pathogenesis [41, 43, 49]. Mattoo et al. demonstrated that CD4^+^ CTL cells which also expressed signalling lymphocytic activation molecule F7 (SLAMF7) were expanded in patients with IgG4-RD, and these cells expressed granzyme B, perforin, IL-1β, TGF-β and IFN-γ, which may be important mediators of tissue damage [43]. Rituximab-induced clinical remission was associated with a reduction in the number of these CD4^+^ CTL, but had minimal or no effect on the frequency and number of CD4^+^GATA3^+^ Th2 phenotype cells or CD4^+^CD25^+^Foxp3^+^ regulatory T cells [49]. The CD4^+^ CTL do not express surface CD20, which adds further support to the hypothesis that CD4^+^ CTL are sustained by cells of B cell lineage [41].

Th1/Th2/Treg cells have been variably implicated in IgG4-RD pathophysiology, but direct evidence for their role is lacking, and Th2 cells may only accumulate in those with IgG4-RD and concomitant atopy [42, 50].

In summary, CD4^+^ CTL may orchestrate IgG4-RD, but be themselves sustained by continuous antigen presentation by B cells or by B cell-dependent growth factors, self-perpetuating an immune response against a specific antigen (whether microbial, environmental or self) [41, 42]. Further characterisation of the pathogenesis of the condition may offer an opportunity for a more rational and targeted therapeutic approach in the future, e.g. elotuzumab, a humanised monoclonal antibody directed against SLAMF7 and approved for patients with relapsed/refractory multiple myeloma [51].

## Neurological manifestations of IgG4-RD

The most common CNS manifestations of IgG4-RD are hypertrophic pachymeningitis and hypophysitis, although direct parenchymal brain involvement and changes secondary to associated vasogenic oedema have been reported as well as inflammatory pseudotumour [52]. IgG4-related neuropathy and IgG4-related perineural disease have been reported, but occur relatively infrequently.

### IgG4-related pachymeningitis

Pachymeningitis secondary to IgG4-RD is a form of hypertrophic pachymeningitis characterised by localised or diffuse inflammation and thickening of the meninges (mostly dura mater)—cerebral, spinal or rarely both [15, 53–55]. Infectious, inflammatory and neoplastic causes need to be considered. Of the inflammatory causes, sarcoidosis, granulomatosis with polyangiitis (GPA), rheumatoid arthritis (RA), Sjögren’s syndrome and IgG4-RD are the most common [15, 54, 55]. The prevalence of hypertrophic pachymeningitis has been evaluated in a Japanese national survey and reported to be 0.949 cases per 100,000, with pachymeningitis due to IgG4-RD being observed in 8.8%, second only to anti-neutrophil cytoplasmic antibody (ANCA)-related pachymeningitis [56]. Hypertrophic pachymeningitis in IgG4-RD was more commonly seen in men (1:0.17), and the mean age of onset was 56.7 years.

Symptoms arising due to hypertrophic pachymeningitis occurring in the context of IgG4-RD typically reflect either focal or widespread meningeal involvement (e.g. hemispheric or basal dura), leading to mechanical compression of structures (e.g. cranial palsies) or to more diffuse symptoms (e.g. headache, seizures and cognitive decline) [55, 57]. Indeed, chronic headache and multiple cranial neuropathies are the most commonly reported symptoms [15, 53, 55, 57, 58]. Symptoms resulting from mechanical compression are dependent on anatomical location. Where there is involvement of the cavernous sinus or superior orbital fissure, possible manifestations include paresis of the cranial nerves II–VI and a combination of retro-orbital pain, altered vision and extra-ocular muscle palsies, such as the Tolosa–Hunt syndrome [15, 54, 59–66]. Involvement of the middle fossa, falcotentorial, cerebellar tentorium and posterior fossa areas typically causes paresis of the cranial nerves VI–XII and/or cerebellar ataxia [15, 53, 54, 67–70]. Lesions in the vertebral canal are more common at the cervical and thoracic levels and may present with radiculopathies, limb paresis and/or sphincter disturbances [54, 59, 71–75].

IgG4-related leptomeningitis has been reported in three patients [14, 76, 77], although the possibility that this may occur due to rheumatoid meningitis rather than IgG4-RD should be considered, particularly given the similarity in histopathology; two patients were known to have concomitant RA and the third had significantly elevated anti-CCP antibodies without clinical features of RA (although detection of anti-CCP antibodies can precede the emergence of clinical features of RA by many years) [15].

In 2014, Lu et al. reviewed 33 cases of biopsy-proven IgG4-related hypertrophic pachymeningitis, and the presenting features were as follows: headache (67%), cranial nerve palsies (33%), visual disturbance (21%, typically diplopia or decreased visual acuity), motor weakness (15%), limb numbness (12%), sensorineural hearing loss (9%), seizures (6%) and cognitive decline (3%) [57]. About 48% had systemic involvement, mostly bone (12%), salivary glands (9%), lungs (9%), kidney (6%), orbital pseudotumour (6%) and retroperitoneal fibrosis (6%). Isolated IgG4-related hypertrophic pachymeningitis was present in 30% of the patients, although, notably, systemic involvement could not be excluded in 27% due to lack of available information.

### IgG4-related hypophysitis

Hypophysitis secondary to IgG4-RD is one of the most recently described forms of hypophysitis and is defined by an inflammatory process of the pituitary gland that can involve contiguous structures [78]. As with AIP, patients with IgG4-related hypophysitis tend to be middle-aged and older men [14, 71, 79–81].

IgG4-related hypophysitis most often presents with panhypopituitarism [81], although anterior hypopituitarism with a combination of hormone deficiencies or diabetes insipidus has also been reported [49, 82, 83]. A predictable constellation of symptoms resulting from pituitary insufficiency may occur (e.g. general malaise, loss of appetite, weight loss, polyuria, polydipsia, amenorrhoea and decreased libido), but neither these nor the symptoms which may arise due to compression of nearby structures are themselves specific to IgG4-RD; alternative underlying aetiologies including Langerhans cell hystiocytosis, sarcoidosis, GPA, infection, neoplasia and iatrogenic causes, such as treatment with interferon or anti-cytotoxic T lymphocyte-associated protein 4 (CTLA-4), need to be considered. However, clues as to the underlying diagnosis may be apparent from the concurrence of systemic features of IgG4-RD, such as retroperitoneal fibrosis, salivary gland disease and lymphadenopathy. Indeed, isolated hypophysitis secondary to IgG4-RD appears to be relatively uncommon, although the limitations of the available data are noted.

Although hypophysitis secondary to IgG4-RD has been considered a rare condition, Bando et al. recently screened 170 consecutive outpatients presenting with hypopituitarism and/or diabetes insipidus and reported that a diagnosis of IgG4-RD was made in 30% with hypophysitis, 22% with hypopituitarism/diabetes insipidus and 4% of all cases of hypopituitarism and/or diabetes insipidus [84]. Bernreuther et al. retrospectively determined the prevalence of hypophysitis secondary to IgG4-RD amongst cases previously diagnosed as primary hypophysitis (lymphocytic hypophysitis, granulomatous hypophysitis and hypophysitis not otherwise specified), and the histological and immunohistochemical analysis of all cases revealed that 41.4% (12/29) of cases previously diagnosed as primary hypophysitis fulfilled the criteria for IgG4-RD [85].

### Inflammatory pseudotumour

Inflammatory pseudotumour is a descriptive term for a family of lesions with diverse aetiology characterised by a tumour-like mass lesion which may mimic neoplastic disease [86]. Histopathological analysis demonstrates hyalinized collagenous tissue admixed with a lymphoplasmacytic infiltrate, composed chiefly of polyclonal plasma cells, lymphocytes and scattered histiocytes with or without eosinophils [86, 87].

The most common anatomical sites for IgG4-related pseudotumours are the orbit, salivary glands, lungs, kidneys, lymph nodes and retroperitoneum. Although involvement of the nervous system appears to be relatively rare, a range of sites have been noted including involvement of the meninges (mimicking meningioma), ventricles, parietotemporal parenchyma, pituitary gland, cranial nerves and spinal cord [87–94]. Symptoms typically arise due to the compressive nature of these lesions and therefore depend on their location.

### Parenchymal brain involvement

**Fig. 1:**
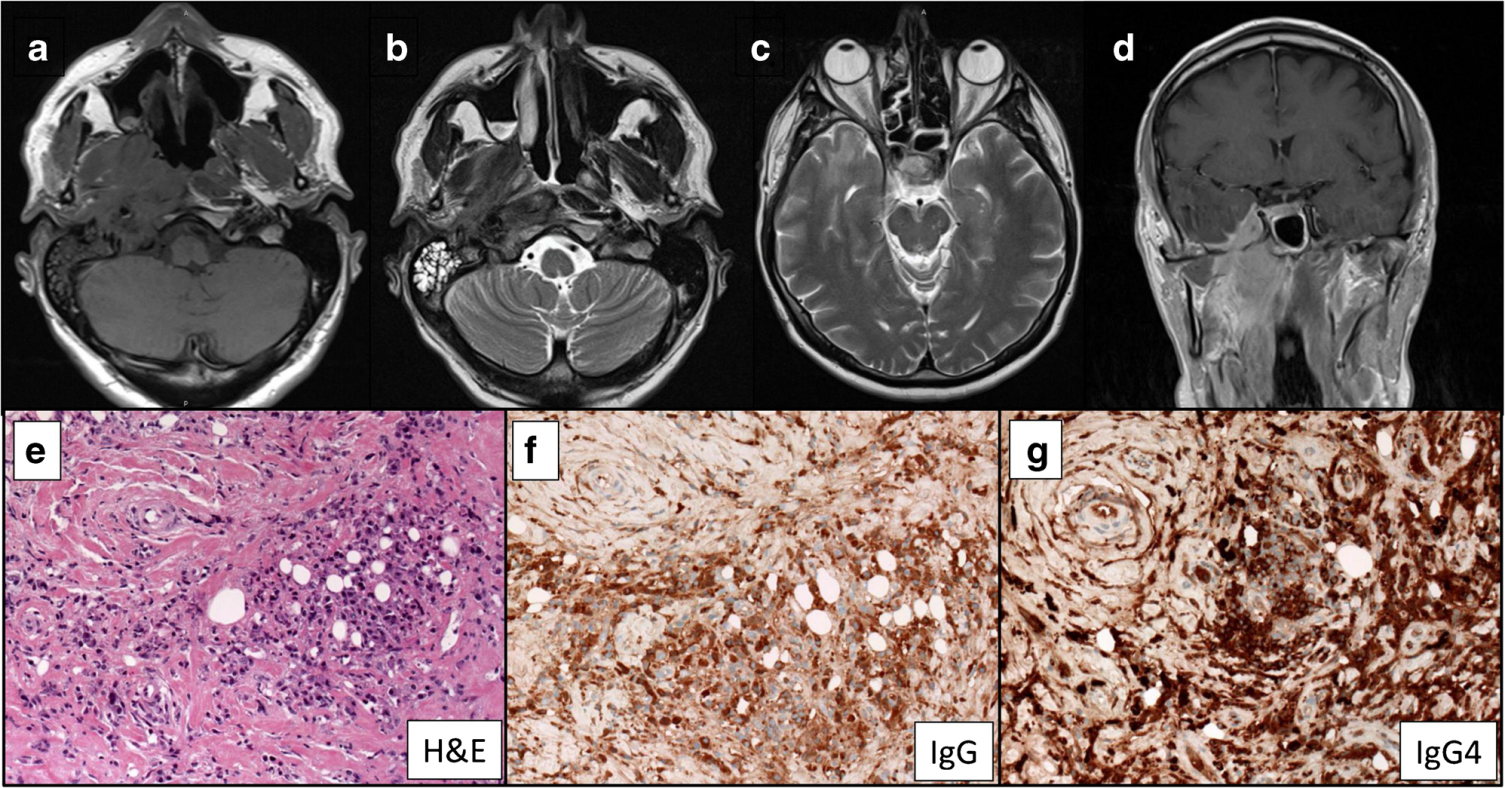
MR imaging (**a**, **b**) demonstrated an enhancing soft tissue mass involving the right posterior nasopharynx with infiltration laterally and posteriorly into the right prevertebral strap muscles and through the pharyngobasilar fascia to involve the medial and lateral pterygoids. The right carotid and internal carotid artery was ensheathed and abnormal tissue was seen in the right carotid canal and jugular foramen. The right cavernous sinus was involved via perineural spread through the foramen ovale. Basal pachy- and leptomeningitis were noted along the floor of the right middle cranial fossa with high signal in the overlying temporal lobe (**c**). Progressive changes were noted in the pterygomaxillary fissure, the muscles of the masticator compartment and throughout the temporalis muscle on the right at the time of representation (**d**). **e**–**g** A diffuse plasma cell-rich, chronic inflammatory cell infiltrate with prominent stromal fibrosis/hyalinization, fat necrosis and focal granulation tissue was evident on biopsy of the anterior temporalis and posterior maxilla (**e**). Immunocytochemistry demonstrated numerous IgG- and IgG4-positive plasma cells (**f**, **g**). Reproduced with permission from Rice et al. [95].

Biopsy-proven parenchymal brain involvement in the absence of pseudotumour formation has been reported infrequently and in association with pachymeningitis or systemic IgG4-RD. Regev et al. reported biopsy-proven involvement of the brain parenchyma in a patient with systemic manifestations of IgG4-RD [52]. Magnetic resonance imaging (MRI) showed multifocal high signal abnormalities on T2 and fluid-attenuated inversion recovery sequences with subtle enhancement with gadolinium. Parenchymal brain involvement is reported in a small number of additional cases, although all were associated with pachymeningitis (Fig. 1) [95–97]. Two had biopsy-proven parenchymal involvement, whilst the other had pachymeningitis secondary to biopsy-proven IgG4-RD with high signal in the overlying temporal lobe. In the latter case, biopsy of the brain parenchyma was not undertaken, and the possibility remains that the imaging abnormalities which were not associated with clinical signs were due to vasogenic oedema rather than direct brain involvement.

## IgG4-related neuropathy and perineural disease

Inoue et al. retrospectively studied 106 patients with IgG4-RD and identified seven with a total number of 21 peripheral nerve lesions [98]. A number of other authors have also reported perineural disease secondary to IgG4-RD [99, 100]. Typically, this has occurred in orbital or paravertebral areas and the lesions have been asymptomatic, indolent and steroid-responsive, although they necessitate differentiation from other potentially aggressive conditions such as lymphoma, neurogenic tumours, sarcoidosis and idiopathic inflammatory pseudotumour. Radiologically, a distinct, perineural soft tissue mass has been reported. Histological analysis of the epineurium has been available in only a limited number of cases, but demonstrates massive lymphoplasmacytoid infiltrate which is rich in IgG4^+^ plasma cells and preferentially involves the epineurium.

To the best of our knowledge, there is only a single report of neuropathy ascribed to IgG4-RD [101]. The authors described a 55-year-old man with histopathologically confirmed IgG4-RD manifesting as mononeuritis multiplex, with electrophysiological findings suggestive of axonal neuropathy. Histopathological analysis of the sural nerve demonstrated marked thickening with abundant collagen fibres and infiltration of IgG4^+^ plasma cells in the epineurium, a moderate degree of myelinated fibre loss, but no evidence of vasculitis. Oral prednisolone was highly effective, with rapid improvement of the neuropathic symptoms.

Recently, Kamiya et al. described an intriguing case of a 68-year-old man who presented with concomitant biopsy-proven IgG4-related sialadenitis and cryoglobulinaemic vasculitis in the context of lymphoma which was in remission [102]. The cryoglobulins were predominantly monoclonal IgG-κ, and biopsies of the skin and sural nerve were more consistent with vasculitis rather than IgG4-RD. The link (if any) between the concomitant IgG4-RD and cryoglobulinaemia remains unclear.

## Diagnostic approach

IgG4-RD is a clinicopathological diagnosis incorporating features from the history and clinical examination, as well as serological, radiological and histopathological investigations. In 2011, diagnostic criteria were proposed and consisted of (1) characteristic diffuse/localised swelling or masses in single or multiple organs; (2) elevated serum IgG4 concentrations; and (3) histopathology demonstrating infiltration of lymphocytes and plasmacytes with demonstration of IgG4^+^ plasma cells (ratio of IgG4^+^/IgG^+^ cells >40% and >10 IgG4^+^ plasma cells/high-power field, HPF) and fibrosis [103]. The diagnosis is deemed definite if all the above features are present, probable if (1) plus (3) are present and possible if (1) plus (2) are present. Yamamoto et al. validated these criteria using a registry cohort that consisted mainly of patients with dacryoadenitis and sialadenitis [104], but they have not otherwise been widely used, as yet.

When confirming the involvement of the nervous system due to IgG4-RD, the approach is similar. However, the diagnostic workup may also include analysis of the CSF; depending on the location of the lesion, there may be greater reticence when considering tissue biopsy, and in some cases with multi-organ involvement, CNS biopsy may not be required. In others, such as those with isolated hypertrophic pachymeningitis, for example, histological analysis is required [57].

Leporati et al. devised specific diagnostic criteria for hypophysitis secondary to IgG4-RD, which permitted diagnosis when MRI of the pituitary was compatible and there was histopathological evidence from another organ or, if that was not possible, increased serum IgG4 levels and prompt response to corticosteroid therapy [82]. These criteria have not been independently verified, but they have been widely used in practice.

Where definitive diagnosis cannot be made, careful consideration of the differential diagnosis must be undertaken. Of these, it is worth considering ANCA-associated vasculitis (particularly GPA) as it shares many of the multisystem features of IgG4-RD, including pachymeningitis and hypophysitis [49, 105, 106]. Furthermore, increased serum levels of IgG4 and infiltrating IgG4^+^ plasma have also been described in GPA [15, 107–109]. Nonetheless, there are some noteworthy differences [49]. The majority of patients with GPA have nasal disease, although some may be subclinical. This may manifest as bloody nasal crusts, nasal septal mucosal erosions and sinus disease. IgG4-RD is less likely to lead to erosive sinonasal disease, usually manifesting with allergic features and, occasionally, nasal masses. In the lungs, GPA more commonly leads to nodular or cavitating lesions, alveolar haemorrhage or bronchial stenosis, and IgG4-RD typically causes pleuritis, nodular pulmonary lesions, ground-glass infiltrates, interstitial fibrosis and/or thickening of bronchovascular bundles. The renal manifestations of GPA and IgG4-RD are also distinguishable as IgG4-RD is characterised by tubulointerstitial nephritis (only rarely by membranous nephropathy) and GPA typically presents with crescentic and necrotizing glomerulonephritis. Serologically, both may be associated with high serum levels of IgG4, but GPA is usually ANCA-positive with antigen specificity for proteinase 3 (less often for myeloperoxidase), and ANCA is typically negative in IgG4-RD. Indeed, although ANCA positivity does not exclude the diagnosis of IgG4-RD, it should prompt the exclusion of a concomitant vasculitic process [105]. Histologically, GPA is usually associated with foci of necrosis, granulomatous inflammation and giant cells, and neutrophils invariably constitute a prominent part of the infiltrate, features not seen in IgG4-RD [110].

Below, we will review components of the diagnostic approach to IgG4-RD, with specific reference to the neurological manifestations of the disease, particularly pachymeningitis and hypophysitis.

### Presentation

None of the neurological manifestations of IgG4-RD are pathognomonic, and they may occur in the context of systemic disease or, less commonly, in isolation.

IgG4-RD usually follows a relapsing–remitting course and may occur, either synchronously or metachronously, in a variety of organs, including the pancreas, bile duct, lacrimal glands, periorbital tissues (e.g. the lacrimal gland and retro-orbital space), thyroid, central and peripheral nervous systems, lung, pleura, heart, pericardium, breast, liver, gastrointestinal tract, kidney, prostate gland, retroperitoneum lymph nodes and skin [1, 2••, 3, 4, 5•, 6, 111–113]. Typically, one organ dominates the clinical picture, and the symptoms are dependent upon the location of the lesions occurring as a consequence of organ swelling with or without compression of nearby structures or tissue damage resulting in loss of function.

IgG4-RD tends to present indolently, with symptoms appearing over months to years [114]. In addition, constitutional symptoms are often subtle or even absent, and patients often feel relatively well even in the setting of multi-organ disease [8, 79, 114]. However, a minority of patients have a more fulminant presentation characterised by constitutional symptoms, fever and multi-organ involvement from the outset [4, 8]. A diffuse array of musculoskeletal symptoms (e.g. arthralgias and enthesopathy) has been reported, although no histopathological abnormalities of the synovium or tenosynovium have been confirmed. A history of atopy is sometimes reported [2••].

### Radiology

Imaging studies in IgG4-RD are useful both for diagnostic and monitoring purposes, although their limitations in the exclusion of other causes of pachymeningitis and hypophysitis have already been highlighted.

On computed tomography (CT) scans and MRI studies, hypertrophic pachymeningitis occurring in the context of IgG4-RD may appear either as a linear dural thickening or as a focal mass, and it may be localised or diffuse [54, 57, 115, 116]. The focal or pauci-focal distribution of changes within the meninges seems to be more frequent in pachymeningitis due to IgG4-RD, although this observation is based on a limited number of patients [15].

On T2-weighted MRI, fibrotic hypertrophic meninges are thickened and relatively hypointense, with foci of hyperintensity being suggestive of inflammation that can be confirmed using gadolinium-enhanced T1-weighted MRI [57, 115, 116]. Conversely, CT scans are more useful in the assessment of concomitant bone involvement, and on CT studies, dural lesions typically appear thickened, hyperdense and contrast-enhanced [57]. In general, MRI is superior to CT for the anatomical evaluation of the optic chiasm, nerve roots, brainstem and skull base, and gadolinium-enhanced T1-weighted MRI studies may also offer superior spatial resolution and facilitate the identification of active inflammation along the dural edges.

Pituitary stalk enlargement is the most common finding on MRI of hypophysitis secondary to IgG4-RD, although there may be isolated involvement of the pituitary, concomitant involvement of the pituitary and stalk, and/or pseudotumour formation [80, 81, 83, 84, 116, 117]. The pituitary lesions are usually hypointense on T2-weighted imaging and show homogeneous contrast enhancement on T1-weighted images [116–123]. Absence of pre-contrast T1 hyperintensity in the posterior pituitary gland may suggest central diabetes insipidus, although this requires confirmation through endocrinological workup [116]. Associated pachymeningitis is occasionally seen [115, 117].

### Nuclear medicine

In recent years, 2-[^18^F]-fluoro-2-deoxy-d-glucose positron emission tomography–computed tomography (FDG PET-CT) has emerged as a potentially useful tool in the diagnosis and monitoring of IgG4-RD [47, 68, 71, 80, 124–128]. In 2014, Ebbo et al. retrospectively analysed 21 patients with IgG4-RD and concluded that FDG uptake is correlated with the disease activity and improves after treatment [124]. The authors also suggested that FDG PET-CT might be more sensitive than conventional radiologic imaging to detect IgG4-RD involvement, although exceptions were reported; a patient with pachymeningitis identified on MRI and two cases with nodular infiltration of the kidney on CT had no detectable abnormality of FDG uptake. These false negative results could be explained by (1) the physiologic fixation of FDG in the brain and kidneys, with failure to detect lesions contiguous to these organs, and/or (2) the limited spatial resolution of PET-CT as the lesion in the ‘missed’ case of pachymeningitis was very small. Others have suggested that, for the evaluation of intracranial meningeal lesions, carbon 11-labelled methionine may be preferable because of its low uptake in the normal brain [129].

Recently, Lee et al. assessed the diagnostic utility of FDG PET-CT in the differential diagnosis of IgG4-RD. The authors compared data from 28 IgG4-RD patients with 66 patients with other diseases (mainly malignancy and inflammatory diseases) [127]. Statistical analysis revealed three variables with greater discriminatory power: maximum standardised uptake value (SUV_max_) of main involved organ (typically mild to moderate and lower than that of other diseases); SUV_max_ of submandibular glands (higher FDG uptake when compared with other diseases); and presence of multi-organ involvement. There was a substantial degree of overlap between the distribution of tissue involvement between IgG4-RD and other diseases, but in this study, FDG PET-CT had a diagnostic sensitivity of 85.7% and a specificity of 66.1% for IgG4-RD.

It is also worth noting that gallium SPECT/CT may also have potential clinical utility in the diagnosis and monitoring of IgG4-RD, particularly given its lower cost and more widespread availability [130].

### Laboratory analyses

Serum inflammatory markers such as the erythrocyte sedimentation rate and C-reactive protein may be modestly elevated in IgG4-RD [114]. Occasionally, a polyclonal gammopathy with high serum IgG and IgE can be found, along with hypocomplementaemia (mostly in the setting of tubulointerstitial nephritis) and peripheral eosinophilia [29, 111, 114].

Elevated serum IgG4 concentrations are characteristic of IgG4-RD. Nonetheless, serum IgG4 has shortcomings both as a diagnostic marker and in monitoring disease activity or predicting disease relapse. For diagnostic purposes, it is neither necessary nor sufficient to confirm a diagnosis of IgG4-RD on its own, although when present it is supportive. Serum IgG4 concentrations tend to be higher in patients with multi-organ involvement [27, 131–135]. However, high serum levels of IgG4 have been reported in healthy individuals and in patients with parasitic diseases, allergic disease, autoimmune diseases including RA, ANCA-associated vasculitis and multicentric Castleman’s disease, as well as certain malignancies (particularly pancreatic), and most of these diseases can be mimickers of IgG4-RD [114, 134, 136–139]. Furthermore, depending on the series, as many as 45% of patients with biopsy-proven IgG4-RD may have normal serum IgG4 concentrations at the time of diagnosis, and this may be of particular relevance to those with isolated hypertrophic pachymeningitis, who may have increased intrathecal IgG4 despite normal serum levels of IgG4 [3, 33, 47, 131, 140].

A recent meta-analysis reported that high serum IgG4 (>135 mg/dL) has a pooled sensitivity of 87.2% and a specificity of 82.6% for the diagnosis of IgG4-RD, although significant heterogeneity was observed [141]. Doubling the cutoff value for IgG4 improved the specificity to 94.8%, at the expense of the sensitivity, which was reduced to 63%. A recent prospective UK cohort found similar diagnostic sensitivity and specificity for serum IgG4, with higher levels being associated with multi-organ involvement and risk of relapse and its levels falling with corticosteroid therapy [134]. In this cohort, IgG4-RD diagnostic criteria were met in only 5.1% (58/1140) of patients who had serum IgG4 measured for the purpose of discriminating IgG4-RD from other disease conditions and in only 22.4% (48/214) of patients who had an elevated serum IgG4. One explanation for some false negative results is the ‘prozone effect’—underestimation of the serum IgG4 concentration in the presence of large antigen excess may occur when nephelometry assays are used, but diluting the samples can prevent this from happening [142]. In the study by Khosroshahi et al., the prozone effect led to falsely low serum IgG4 concentrations in 26% of patients tested, and this effect was more likely to occur in patients with active disease [142]. Therefore, one must not rely exclusively on serum IgG4 levels to diagnose IgG4-RD.

The ability of serum IgG4 levels to predict relapses and effectively monitor treatment response is controversial—for example, in the study reported by Kamisawa et al., the relapse rate was 30% in those with persistently elevated IgG4 levels and 10% in those with normal concentrations [143, 144].

More recently, Wallace et al. showed that circulating plasmablasts are elevated in active IgG4-RD (even in patients with normal serum IgG4 concentrations) and that plasmablast counts are a potentially useful biomarker for diagnosing IgG4-RD and assessing its response to treatment [44]. Measurements of peripheral blood plasmablasts may be particularly useful for those patients with normal serum IgG4 concentrations but high clinical suspicion for active IgG4-RD, especially if a biopsy is not feasible and appropriate measures have been undertaken to exclude malignancy [44]. Although, intuitively, this approach may have particular advantages for the subset of patients with isolated neurological manifestations of IgG4-RD, there are no data available to confirm or refute this.

### CSF analysis

CSF analysis is of particular importance with regard to IgG4-RD, not only because it offers the possibility to make a positive diagnosis of disease mimics and may give specific information about intrathecal synthesis of IgG4. CSF evaluations in patients with pachymeningitis secondary to IgG4-RD generally reveal clear fluid with normal glucose concentration, normal to mildly increased protein levels and a variable degree of lymphocytic pleocytosis [15, 145]. However, such findings are not specific and cannot differentiate pachymeningitis secondary to IgG4-RD from other forms. The first reports of intrathecal IgG synthesis with an oligoclonal pattern and prominent intrathecal IgG4 production (high IgG4 level and IgG4 index) were from Della-Torre et al. [47, 48]. These authors described the CSF findings of three patients with pachymeningitis secondary to IgG4-RD. All had oligoclonal bands with high IgG4 index at baseline, which, where examined, normalised after treatment. Subsequently, Della-Torre et al. compared the findings of these three patients with nine controls and 21 patients with hypertrophic pachymeningitis due to alternative causes and concluded that quantification of CSF IgG4 may be a diagnostic tool, particularly when serum and CSF IgG4 concentrations were interpreted in relation to the blood–CSF barrier [145].

### Histopathology

Definitive diagnosis of IgG4-RD requires an appropriate histological appearance with increased numbers of IgG4^+^ plasma cells (or an elevated IgG4/IgG ratio) in tissue [5•, 14, 110]. The key morphologic features of IgG4-RD histology are the following: (1) a dense lymphoplasmacytic infiltrate; (2) fibrosis that is organised in a storiform pattern; (3) obliterative phlebitis; and a (4) mild-to-moderate eosinophil infiltrate [5•, 110]. In the majority of cases, these include a dense lymphoplasmacytic infiltrate and storiform-type fibrosis, but exceptions to this rule exist. In organs such as the lymph node, kidney, and the salivary and lacrimal glands, storiform-type fibrosis or obliterative phlebitis may be inconspicuous or absent [5•]. The most specific histological finding seems to be obliterative phlebitis, although this appears not to be particularly prominent in neurological IgG4-RD [14, 15, 84, 85].

The inflammatory lesion frequently forms a tumefactive mass that may destroy the involved organ, and the inflammatory infiltrate is composed of a mixture of T and B lymphocytes. T lymphocytes predominate in the infiltrate and are usually present diffusely, whilst B cells tend to be located within lymphoid aggregates or even germinal centres [5•]. Semiquantitative analysis of IgG4 immunostaining typically reveals the presence of more than 10 IgG4^+^ plasma cells/HPF or a ratio of IgG4^+^ plasma cells to IgG^+^ plasma cells higher than 40%, but individualised cutoffs based on the site of involvement may be required [5•, 110, 135]. For both pachymeningitis and hypophysitis due to IgG4-RD, it is generally accepted that the standard cutoff of 10 IgG4^+^ plasma cells per HPF is reasonable [15]. A lower cutoff point for IgG4^+^ cells may be acceptable in cases with the characteristic morphologic features [2••]. IgG4-RD is more difficult to diagnose in the late phase of organ involvement, when fewer plasma cells are present and fibrosis predominates (e.g. the retroperitoneum and meninges)—the pattern of fibrosis and the ratio of IgG4 to total IgG provide crucial information in this context [135]. It must also be remembered that infiltration of tissues with IgG4^+^ plasma cells is not specific for IgG4-RD and should always be interpreted in light of the accompanying clinical, histological, radiological and serological findings [110].

## Treatment

Because of its rarity, treatment of neurological disease secondary to IgG4-RD has been extrapolated from the relatively limited evidence available for even the more common organ manifestations. No randomised clinical trials have been performed in IgG4-RD, and the best evidence for therapeutic options comes from systematic review of the literature (Brito-Zeron et al., for example) [9•] and expert guidance [10••].

Not every patient with IgG4-RD needs treatment, and given that some cases of spontaneous remissions have been reported, a ‘watchful-wait’ decision may be appropriate in some patients (e.g. asymptomatic lymphadenopathy) [2••, 9•, 144, 146–148]. However, when vital organ involvement is present, it usually requires aggressive and immediate treatment to prevent organ dysfunction and failure [2••, 9•, 41]. In 2015, an International Consensus Statement on the Treatment of IgG4-RD recommended that all patients with symptomatic, active disease require treatment [10••]. Increasingly, it is recognised that a major determinant of treatment responsiveness is the degree of fibrosis within the affected organs; untreated IgG4-RD often progresses from lymphoplasmacytic inflammation to extensive fibrosis, and at this stage, patients are less likely to have a response to treatment [2••, 29, 148, 149].

Glucocorticoids are the first-line treatment, unless there is a contraindication [10••, 147]. Patients with IgG4-RD (including those with neurological involvement) usually have an excellent but often unsustained clinical response to glucocorticoids [1, 8, 15, 29, 81, 82, 112, 143, 144, 150]. Responsiveness to glucocorticoids is characteristic early in the disease course, before the onset of significant tissue fibrosis ensues. In this respect, FDG PET-CT may have an important role in defining the extent of the inflammation. Clinical responses to glucocorticoids are usually quick (within 2 weeks) and are typically accompanied by a decline in serum IgG4 concentration [29, 140, 143, 144, 148]. However, this serological response to glucocorticoids is not specific for IgG4-RD and should not be used to differentiate it from other conditions [134].

Relapse rates after steroid withdrawal are high, and may also be significant during glucocorticoid taper or maintenance therapy [143, 144, 151, 152]. A recent systematic review by Brito-Zéron et al. reported that treatments with first-line glucocorticoid regimens were 97% effective, but carried a 33% risk of relapse [9•]. Typically, relapses were themselves responsive to glucocorticoid treatment, albeit at increased doses.

For remission induction of IgG4-RD, prednisolone at a dose of 30–40 mg/day is commonly instigated [153]. However, for neurological manifestations of IgG4-RD (mostly pachymeningitis), an initial course of intravenous methylprednisolone (e.g. 500 mg–1 g for 3 days) has been used in an attempt to rapidly and effectively dampen the inflammatory response and prevent irreversible CNS damage [47, 65, 71, 81, 112, 154], although some authors have reported positive outcomes with relatively low doses of glucocorticoids in pachymeningitis [96] and replacement therapy in hypophysitis [80, 81, 121, 128, 155, 156].

The dose and duration of ongoing glucocorticoid therapy are a matter of ongoing debate. For example, some clinicians from Asia recommend treatment with prednisolone 0.6 mg kg^−1^ day^−1^ for 2–4 weeks, followed by a tapering schedule over a period of 3–6 months down to a maintenance dose (2.5–5 mg/day), which is then maintained for up to 3 years. These recommendations are mostly based on a study conducted by Kamisawa et al. in AIP—23% of the patients on glucocorticoid maintenance treatment relapsed versus 34% of the patients off treatment [143]. Relapse occurred within 6 months after starting treatment in 32%, within 1 year in 56% and within 3 years in 92%, leading the authors to suggest prolonging maintenance treatment for 3 years. These observations have not been reproduced in other studies, and taking into account the morbidity associated with long-term steroid therapy and the steroid responsiveness of relapse, some authors advocate discontinuation of glucocorticoid treatment after 3–6 months of treatment [152, 157–159]. Nonetheless, certain patients do seem to benefit from maintenance of glucocorticoid therapy, although the optimal dose and duration of the treatment are uncertain [10••]. There is some evidence to suggest that patients with multi-organ disease, significantly elevated serum IgG4 concentrations, involvement of the proximal bile ducts or a history of disease relapse are at higher risk of early recurrence [144, 159]. Patients with organ-threatening IgG4-RD (including hypophysitis and pachymeningitis) may also benefit from long-term maintenance therapy in an effort to minimise irreversible CNS damage [10••, 83].

Azathioprine, mycophenolate mofetil, methotrexate (MTX), 6-mercaptopurine, tacrolimus and cyclophosphamide have been used as potential ‘steroid-sparing agents’ (SSA) or remission maintenance drugs (especially after relapse), but their long-term efficacy requires further evaluation [8, 9•, 10••, 144, 150–152, 157–160]. MTX was specifically assessed in a recent retrospective small study by Della-Torre et al. and was reported to be effective in maintaining clinical and serological responses induced by glucocorticoids, enabling a substantial reduction in the overall dose of steroids in all patients and their withdrawal in six of ten patients within 6–12 months [161]. None of these patients had neurological disease secondary to IgG4-RD, and it should be noted that others have reported conflicting results of SSA use (predominantly MTX) in neurological disease caused by IgG4-RD [15, 63, 64, 69–71, 83, 118, 162, 163].

For those patients with IgG4-RD that is either refractory to glucocorticoids or which relapses, B cell depletion with rituximab (RTX) appears to represent a promising treatment [133, 149, 160, 164–166]. Patients treated with RTX (typically two 1-g intravenous infusions given 2 weeks apart) have generally demonstrated prompt clinical, radiological and serologic responses, enabling rapid glucocorticoid taper and leading to a swift decline in serum IgG4 [149, 160, 164, 166]. Moreover, the decline in serum IgG4 appears to be more pronounced than the other IgG subclasses, suggesting that one potential mechanism of RTX efficacy in IgG4-RD is through interference with the repletion of short-lived plasma cells that are producing IgG4, although it also appears to be efficacious in the group of patients with normal serum levels of IgG4 [133, 149, 164, 166]. RTX has been shown to be effective in controlling pancreatic and extra-pancreatic IgG4-RD in two recent studies [133, 166]. One was a prospective single-arm open-label trial and included patients with multisystem disease, although none had neurological involvement (NCT01584388) [133]. The other was a retrospective cohort study that also included patients with multisystem disease, but only 2 of 60 patients had CNS disease [166].

The prospective trial was conducted in a high-risk group of patients (multi-organ involvement, prior relapse, previous exposure to steroids and failure of previous SSA), and the results are reassuring; 97% responded, with response generally observed after 2 weeks and sustained for 6 months. At 6 and 12 months after RTX, only 10% of the participants were on glucocorticoid treatment, thereby avoiding steroid-related adverse effects in a population of patients that may be particularly vulnerable. Retreatment with RTX for relapses was deemed necessary in 13% (4/30) during the 12-month period after enrolment. In the retrospective cohort, clinical response was seen in 95% of the patients, but 37% relapsed following successful treatment [151]. Predictors of disease relapse were high baseline values of IgG4, IgE and the total eosinophil count. Additional reports have emerged of RTX being effective in a number of cases of IgG4-RD affecting the CNS, generally pachymeningitis [15, 61, 66, 70, 112, 163].

Serial treatments with RTX (e.g. 1 g every 6 months) may lead to progressive decline in serum IgG4 concentrations and better disease control, although this observation needs confirmation [133, 149, 160, 167] and the possibility that patients who do not benefit from RTX are underreported should be considered.

Apart from pharmacological therapy, some patients may need urgent surgical intervention (e.g. laminectomy) in the face of ongoing neurological symptoms (e.g. paraparesis), and this has been more frequently reported in spinal pachymeningitis. In fact, some of these patients have been treated with surgery alone, without concomitant glucocorticoid therapy [58, 168].

To assess the response to treatment, there is general agreement that one should use a combination of clinical, radiological and serological findings [10••]. An IgG4 responder index has been developed that takes into account disease activity across a full spectrum of potential organ involvement, the serum IgG4 concentration, the need for treatment on an urgent basis, the recording of damage in organ systems and the cumulative steroid dose over the preceding 28 days, but whilst the potential utility of such a score is acknowledged, this particular tool requires validation [169].

## Conclusion

IgG4-RD is a systemic disease of unknown cause that affects virtually every organ system, and neurological manifestations have been increasingly recognised and reported. The most common of these are hypophysitis and pachymeningitis, which may be life-threatening, although treatable. Therefore, the differential diagnosis of IgG4-RD must be considered when a patient presents with signs or symptoms suggestive of hypophysitis or pachymeningitis, and special attention should be placed to other clues that may suggest IgG4-RD (e.g. retroperitoneal fibrosis, sialadenitis and dacryoadenitis). Glucocorticoids are usually effective in the induction of remission, although a number of patients relapse. In this regard, retreatment with glucocorticoids and/or a SSA or RTX is usually necessary, although the optimal treatment strategy remains uncertain. There is a pressing need for clinical trials to address this as the long-term side effects of glucocorticoids add significant morbidity. In the future, new insights derived from a more complete understanding of the pathogenesis of IgG4-RD may facilitate the development of more effective and better targeted pharmacological options.
